# Nonfatal opioid overdoses before and after Covid-19: Regional variation in rates of change

**DOI:** 10.1371/journal.pone.0263893

**Published:** 2022-03-09

**Authors:** Albert J. Burgess-Hull, Kirsten E. Smith, Leigh V. Panlilio, Destiny Schriefer, Kenzie L. Preston, Aliese Alter, Christopher Yeager, Timothy Chizmar, Ted Delbridge, Kenan Zamore, Jeff Beeson, David H. Epstein

**Affiliations:** 1 National Institute on Drug Abuse Intramural Research Program, Baltimore, Maryland, United States of America; 2 Office of National Drug Control Policy, High Intensity Drug Trafficking Area, Washington, DC, United States of America; 3 Maryland Institute for Emergency Medical Services Systems (MIEMSS), Baltimore, Maryland, United States of America; 4 District of Columbia Department of Health, Washington, DC, United States of America; Technion - Israel Institute of Technology, ISRAEL

## Abstract

**Background:**

The Covid-19 pandemic and its accompanying public-health orders (PHOs) have led to (potentially countervailing) changes in various risk factors for overdose. To assess whether the net effects of these factors varied geographically, we examined regional variation in the impact of the PHOs on counts of nonfatal overdoses, which have received less attention than fatal overdoses, despite their public health significance.

**Methods:**

Data were collected from the Overdose Detection Mapping Application Program (ODMAP), which recorded suspected overdoses between July 1, 2018 and October 25, 2020. We used segmented regression models to assess the impact of PHOs on nonfatal-overdose trends in Washington DC and the five geographical regions of Maryland, using a historical control time series to adjust for normative changes in overdoses that occurred around mid-March (when the PHOs were issued).

**Results:**

The mean level change in nonfatal opioid overdoses immediately after mid-March was not reliably different in the Covid-19 year versus the preceding control time series for any region. However, the *rate of increase* in nonfatal overdose was steeper after mid-March in the Covid-19 year versus the preceding year for Maryland as a whole (B = 2.36; 95% CI, 0.65 to 4.06; p = .007) and for certain subregions. No differences were observed for Washington DC.

**Conclusions:**

The pandemic and its accompanying PHOs were associated with steeper increases in nonfatal opioid overdoses in most but not all of the regions we assessed, with a net effect that was deleterious for the Maryland region as a whole.

## Introduction

In January 2020, the United States experienced initial rises in severe acute respiratory syndrome coronavirus 2 (SARS-CoV-2; hereafter referred to as “Covid-19”) infections [1–3]. By March, state and local government began issuing public-health orders (PHOs) such as school closures, limitations on commerce and gatherings, and stay-at-home orders [4–6]. The potential impact these changes have had on problematic use of psychoactive drugs and accompanying rates of overdose has been of particular concern [7–10]. Before the Covid-19 pandemic, the US was already experiencing multiple drug epidemics (e.g., opioids, alcohol, amphetamines, novel synthetic drugs), with opioids being the primary drivers of overdose [11, 12]. Understanding the ways that Covid-19 and its accompanying PHOs have impacted opioid overdoses could inform the design and implementation of preventive measures during future public-health emergencies.

Since the start of the Covid-19 pandemic, reports of changes in opioid or other drug-related overdoses during Covid-19 have focused mainly on fatal overdose. Non-peer reviewed reports from state and national public-health agencies have almost all documented outbreaks or prolonged increases in opioid and other drug-related fatalities since the start of Covid-19 [13]. Peer-reviewed reports have also detailed an increase in fatal opioid overdoses, either in aggregate during 2020, or after the implementation of Covid-19 PHOs [e.g., 14–16].

Patterns of nonfatal overdoses are generally less documented through official channels, but recently there has been a concerted effort by ODMAP (http://www.odmap.org)—a overdoses and to use this information for public health and safety. Identifying trend changes in nonfatal overdose might be important as a bellwether not only of fatality but of the many deleterious effects associated with increased opioid use in a region. Furthermore, nonfatal overdoses are not simply a dodged bullet. The hypoxia that accompanies an opioid overdose can be prolonged and severe, with chronic and possibly lifelong sequelae that may accumulate across multiple nonfatal overdoses during a lifetime [17]. Perhaps most importantly, the risk for fatal overdose often increases after a nonfatal overdose [18–20].

To our knowledge, only a few peer-reviewed reports have examined changes in nonfatal overdoses separate from fatal overdoses during Covid-19. These studies, which examined changes in Emergency Department (ED) visits for nonfatal overdoses in a small sample of healthcare systems, found increased *rates* of such visits (as a proportion of all ED visits) during 2020 compared with previous years [16, 21, 22]. However, *counts* of such visits increased in only some of the health systems evaluated, and even declined in others [22]. Recent data released by the CDC also documented variation across US states in suspected nonfatal overdoses from ED visits [23]. These findings suggest that changes in nonfatal overdoses during Covid-19 are not entirely straightforward: at least for ED visits for nonfatal overdoses, there appears to be between-state variation in these changes. State health authorities may also be interested in such variability *within* states and from additional data sources given the overall paucity of data.

To address this gap, we used near real-time data from ODMAP to examine regional differences in the impact of Covid-19 PHOs on suspected nonfatal overdoses in Washington DC, Maryland, and Maryland state’s five geographical regions. To prevent erroneously concluding that changes in nonfatal overdoses after the PHOs in 2020 were the specific result of those PHOs rather than normative seasonal patterns, we used interrupted time series analysis with a historical time series of comparable overdose counts from the same regions. Finally, we focused on estimating both the immediate potential changes in nonfatal overdoses after PHO implementation, and any enduring changes in the longer-term trend of overdoses in the different geographical regions.

## Methods

### Data sources

These analyses were the result of a collaboration agreement between investigators at the National Institute on Drug Abuse’s (NIDA) Intramural Research Program (IRP) in Baltimore, Maryland and the deputy director of the Washington/Baltimore High Intensity Drug Trafficking Area program’s (HIDTA) Overdose Detection Mapping Application Program (ODMAP) data-aggregation project. ODMAP is an online platform for data collection, visualization, and reporting of suspected overdoses. It was launched as a pilot program in parts of Maryland and West Virginia in early 2017; it now receives data from states throughout the US, although we have thus far only received permission to analyze data from Maryland (MD) and Washington DC. Suspected overdose events are uploaded to ODMAP from participating public-health or law-enforcement agencies manually or through an API. Users can visualize suspected overdoses that have been entered into the database via an online dashboard and run simple analyses. The overall goal of ODMAP is to provide near-real-time surveillance of suspected overdose events across different regions in the US. More information on ODMAP can be found in the S1 Appendix.

Suspected nonfatal overdoses in which opioids were recorded as the primary contributor (whether or not alcohol or other drugs were involved) were uploaded to ODMAP from the Maryland Institute for Emergency Medical Services Systems (MIEMSS) and DC Health (Washington DC). Per Maryland state legislative mandate, MIEMSS is required to submit geotagged data on suspected overdoses to ODMAP within 24 hours of responding to an incident. DC Health data are uploaded to ODMAP from an EMS data repository in real time (on average within 15 minutes of the closure of a patient-care record). Because MIEMSS and DC Health started sharing data with ODMAP at different times, the timeframes of overdose data were slightly different for the two regions: July 1, 2018 to September 10, 2020 for Maryland, and August 6, 2018 to October 25, 2020 for Washington DC (DC).

Before providing the data to NIDA, HIDTA completed a data-use agreement with MIEMSS and DC Health. Because no personal identifiers were associated with the data received by the NIDA coauthors, this project was exempted from IRB review by the NIH IRB office.

### Outcomes

#### MIEMSS data

MIEMSS’s initial inclusion criterion for submitting a report to ODMAP was any case where a patient was administered naloxone by Emergency Medical Services (EMS) or prior to EMS arrival. Reports also included the approximate address where the patient was initially encountered or where the overdose occurred, along with the date and time. On July 1, 2019, MIEMSS updated its methods for defining overdose incidents to rule out non-opioid-related overdoses more accurately. The new definition restricted reports to EMS cases where naloxone was administered and there was either: (a) a primary impression of “Suspected opioid overdose (ICD-10-CM F11.9)” or “Poisoning/Overdose/Drug Abuse (ICD-10-CM T50.90)” [24], or (b) a positive response to a service-defined question ("Do you think this patient is suffering from an opioid overdose?"). By MIEMSS estimates, the definition change resulted in a reduction of overdose counts of approximately 14.5%. The additional reports from the naloxone-only definition likely represent cases in which naloxone was administered, but there was uncertainty whether opioids contributed to the patient’s presentation. To adjust for this, we subtracted 14.5% from all Maryland overdoses prior to July 2019. In sensitivity analyses to examine the robustness of the ITS models to MIEMSS estimates, we found that even large misspecifications in these estimates (e.g., > 10%) did not appreciatively change our major conclusions (see S1 Appendix for sensitivity analyses and additional details on the definition change).

#### DC health data

Standardized case definition of a suspected nonfatal overdoses follows the National Emergency Medical Services Information System (NEMSIS) guidance. A nonfatal opioid overdose is defined as any eligible 911 response where: (a) the Provider’s Primary Impression or Provider’s Secondary Impression are opioid overdose related, or (b) the Primary Symptom or Other Associated Symptoms are opioid overdose related, or (c) medication Administered is naloxone or Narcan and Response to Medication Administered is improved. The patient care report narrative is also queried for opioid- and overdose-related keywords to validate previously identified incidents or identify incidents not previously included. Reports also included the approximate address where the patient was initially encountered or where the overdose occurred, and the date and time.

### Study design

We used an interrupted-time-series design (ITS) [25, 26] to compare changes in nonfatal overdoses before and after the implementation of Covid-19 public health orders (our defined *intervention*). Although ITS analysis of a single time series is considered one of the strongest quasi-experimental approaches [25], it can be further strengthened by the inclusion of a control time series to help rule out cyclical or ongoing influences (e.g., increases or decreases in an outcome that repeat yearly at a specific time) coinciding with the intervention [27]. We took that approach by including a “no-PHO” historical control time series (MD: July 2, 2018 to September 2, 2019; DC: August 6, 2018 to October 20, 2019) in all of our models in addition to the time series of interest, which we called the “Covid year” (MD: July 1, 2019 to September 7, 2020; DC: August 5, 2019 to October 25, 2020). The differences in the timeframes for the two datasets were due to differences in the dates MIEMSS and DC Health started sharing data with ODMAP. The Covid year contained the implementation of PHOs, which, in Maryland, came into effect on March 5, 2020 (state of emergency) and March 30, 2020 (stay-at-home orders). In DC, these orders came into effect on March 11, 2020 (state of emergency) and March 30, 2020 (stay-at-home orders). With the inclusion of the historical control time series, we adjusted for changes in nonfatal overdoses that occurred around mid-March in a year when the Covid-19 pandemic and its accompanying PHOs did not occur. If patterns during the Covid year showed changes similar to those found in the no-PHO historical series, this would weaken the case for a causal connection between Covid PHOs and overdose rates.

To examine regional differences within Maryland, we split the data into five regions: Western, Capital, Central, Southern, and the Eastern Shore. The counties that compose these regions are listed in eTable 1 of the supplemental material S1 Appendix. We used this regional division because it is generally accepted as reflective of the state’s geographic and economic demarcations (Maryland Marketing Partnership, 2021) and because smaller subdivisions (such as counties) would have resulted in sparse data for some regions. These regions are also differentiated by factors associated with regional variation in overdose numbers: population density, income, and EMS request for services (Forati, Ghose, & Mantsch, 2021 [28]; Haffajee et al., 2019 [29]; Monnat, 2019 [30]). Supplemental eTable 2 in S1 Appendix displays median household income and population estimates for the five regions for the 2019 calendar year.

### Data analyses

The daily counts of nonfatal overdoses were aggregated to weekly counts to reduce noise. We then used segmented linear regression to fit ITS models. Although Maryland and DC declared a state of emergency (SOE) at different times in early March, both regions implemented stay-at-home orders on the same day (March 30, 2020). We made the assumption that changes (e.g., disruption to addiction treatment services, changes to illicit drug supplies, loneliness/boredom, childcare/economic burden) related to the initial SOE PHO would have delayed effects on overdose rates, and therefore selected March 31, 2020 as the intervention “change point.” We refer to this intervention change point generically as AfterMarch. We were interested in estimating changes in nonfatal overdose before and after this timepoint.

Each model included a constant term (intercept), a binary indicator to assess whether the mean count of overdoses changed after March (AfterMarch: 0,1), a linear slope term (WeekSlope) quantifying overdose counts from July (or August for DC) through March (i.e., when AfterMarch = 0), an interaction term quantifying the slope change in March, a binary indicator for Covid year versus control year, and two more interaction terms, which were the terms of primary interest. The first interaction term (CovidYear X AfterMarch) tested whether the *mean level* change in overdose counts before and after March differed for the control year versus the Covid year. This allowed us to test whether there was an immediate change in nonfatal overdoses after PHOs and whether this change differed from the control timeframe. The second interaction term (CovidYear X AfterMarch X WeekSlope) tested whether the change in the *slope* of weekly overdoses before and after March differed for the control year versus the Covid year. This allowed us to test whether there was a *sustained* change in nonfatal overdoses and whether this changed differed from the control timeframe. We examined evidence for an immediate and sustained change because we hypothesized that changes related to PHOs might have both an immediate impact (e.g., due to closures in addiction treatment clinics) and longer-term impact on counts of overdoses.

We fit separate models to examine changes in nonfatal overdoses for Maryland as a whole and for Washington DC. For finer geographical granularity within Maryland, we fit a separate model for each of the five regions. All models used Newey-West heteroskedasticity-and-autocorrelation-consistent standard errors [31]. We also tested the inclusion of quarter-yearly indicator variables to adjust for seasonal changes in overdoses. These indicator variables only improved the relative fit of the Washington DC models as measured by the AIC and BIC. Thus, we included these indicators in the Washington DC models and excluded them from all Maryland models. Alpha was set at .05, two-tailed. All analyses were conducted using the R (version 3.6.2) programming language (R Core Team, 2019 [32]).

## Results

### Overall trends in nonfatal-overdose rates for Maryland and Washington, DC, 2018–2020

**Fig 1:**
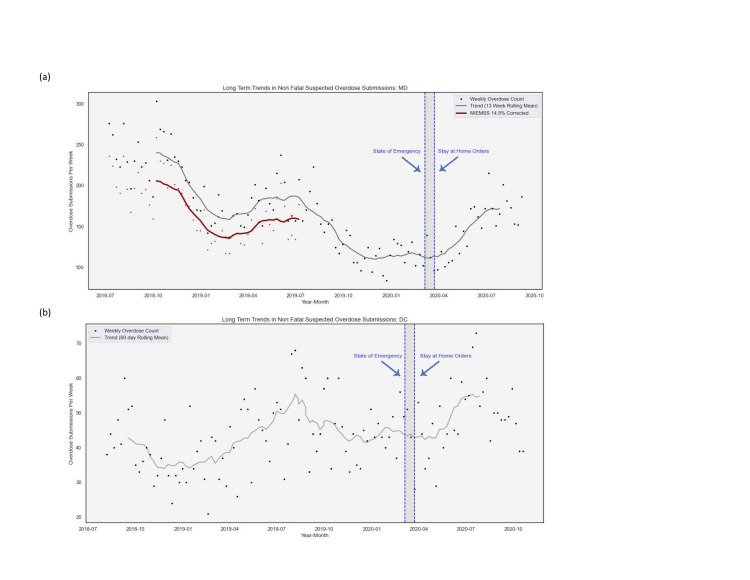
Weekly counts of suspected nonfatal overdoses in Maryland state and Washington DC from 2018–2020. (a) Maryland State. (b) Washington DC. Dashed lines represent the official declaration of a state of emergency (MD: March 5th, 2020; DC: March 11th, 2020) and stay at home orders (MD and DC: March 30th, 2020).

The unadjusted weekly counts of nonfatal overdoses for the entire analysis period (July 2018 to September 2020) are shown in Fig 1. Even though data have been aggregated from daily to weekly totals, variability across weeks remained prominent. Longer-term trends predating the Covid-19 pandemic are also apparent. Specifically, nonfatal overdoses in Maryland were declining over the entire period prior to Covid-19, with a levelling out around November 2019, followed by a distinct increase starting around April 2020. In contrast, nonfatal overdoses in DC appeared to be generally increasing prior to Covid-19.

### Changes in nonfatal-overdose rates with Covid-19 PHOs

**Fig 2:**
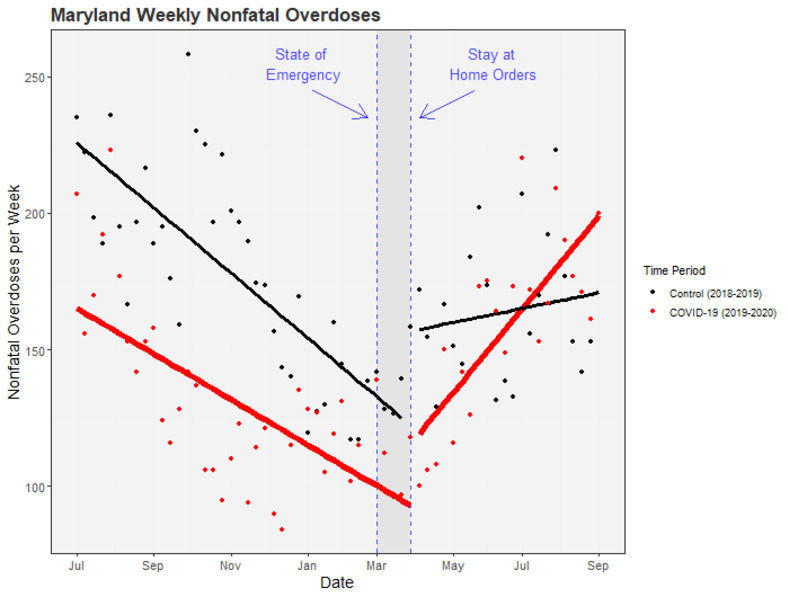
ITS adjusted weekly nonfatal overdose counts in Maryland state pre- and post-Covid-19 public health orders. Points represent weekly counts of suspected nonfatal overdoses. Solid lines represent ITS-model-predicted counts: red for the Covid-19 time series (including March 2020), black for the preceding time series (including March 2019). Dashed horizontal lines mark the official declaration of a state of emergency (March 5th, 2020) and stay-at-home orders (March 30th, 2020) in Maryland state.

**Fig 3:**
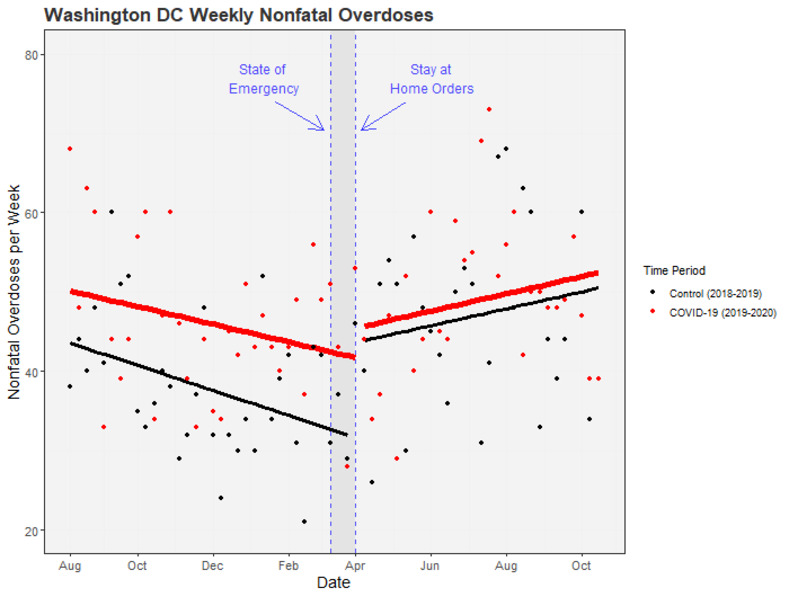
ITS adjusted weekly nonfatal overdose counts in Washington DC pre- and post-Covid-19 public health orders. Points represent weekly counts of suspected nonfatal overdoses. Solid lines represent ITS-model-predicted counts: red for the Covid-19 time series (including March 2020), black for the preceding time series (including March 2019). Washington DC models are adjusted for seasonality with quarter-year dummy variables. Quarter-year dummies are set to their means to generate a smooth line. Dashed horizontal lines mark the official declaration of a state of emergency (March 11th, 2020) and stay-at-home orders (March 30th, 2020) in Washington DC.

**Fig 4:**
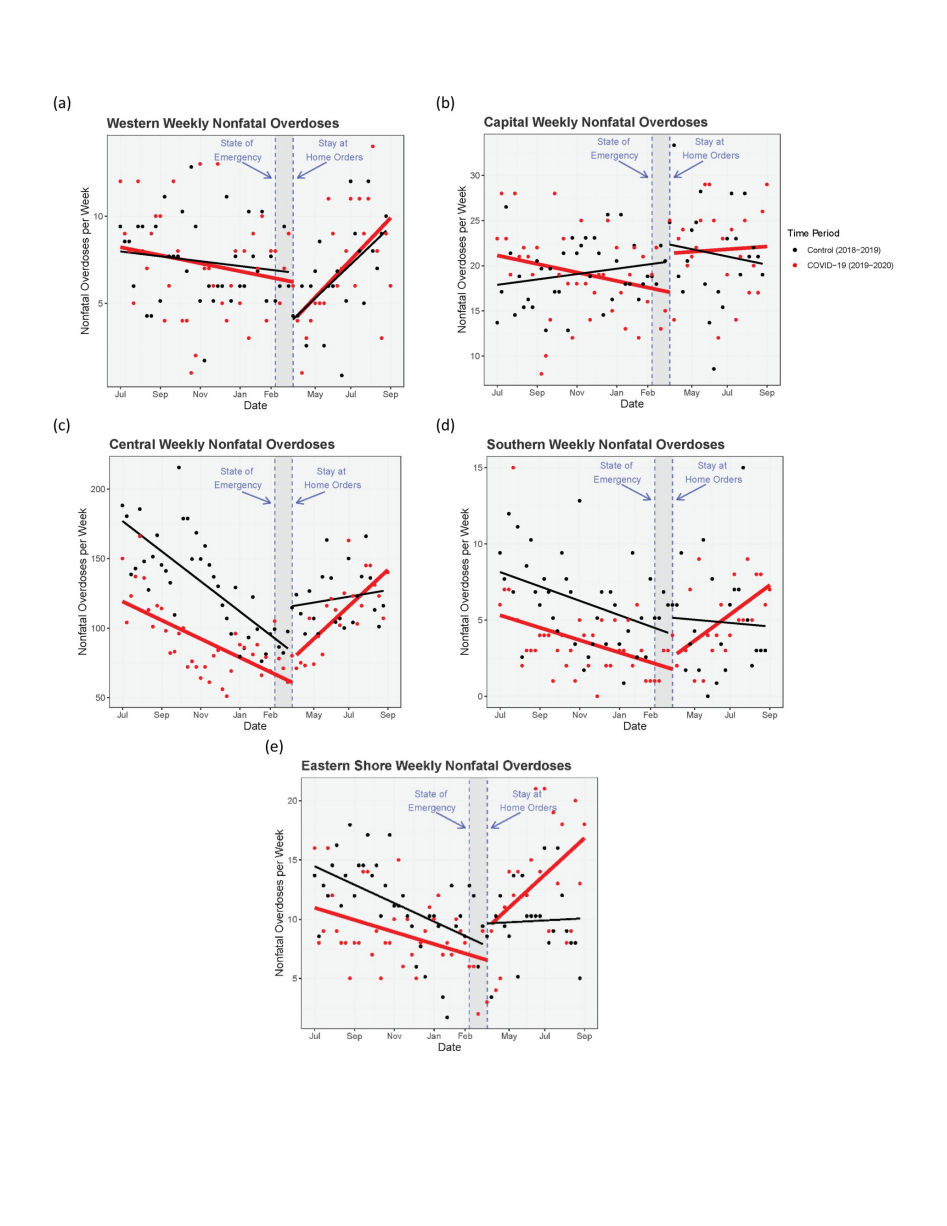
ITS models of weekly nonfatal overdoses for regions within Maryland. Regions in Maryland state: Western (a), Capital (b), Central (c), Southern (d), and Eastern Shore (e). Points represent weekly counts of suspected nonfatal overdoses. Solid lines represent ITS-model-predicted counts. Dashed lines represent the official declaration of a state of emergency (March 5th, 2020) and stay at home orders (March 30th, 2020) in Maryland.

The ITS results are summarized in Table 1 and are shown graphically in Figs 2–4. We also include unadjusted mean weekly counts of overdoses before and after March for each region. Full statistical output from the ITS models is in the Supporting Material S1 Appendix.

**Table 1:** Mean weekly nonfatal overdose counts and key interrupted time series findings for Maryland state models, Maryland regional models, and Washington DC models.

			Immediate Impact of PHOs?			Sustained Impact of PHOs?			
	Mean weekly count: nonfatal overdoses		Difference in *mean level change* before vs. after March for control vs. Covid year^a^			Difference in *weekly slopes* before vs. after March for control vs. Covid year^b^			Key Findings
Region	Before March	After March	*B*	95% CI	*p*	*B*	95% CI	*p*	
**MD State**			-9.21	-47.56 to 29.15	.635	2.36	0.65 to 4.06	.007	Similar pre-March decrease both years; Steeper post-March increase in 2020
Covid timeseries^c^	129.05	159.18							
Control timeseries^d^	175.41	164.15							
**MD: Western**			0.73	-2.80 to 4.27	.682	0.05	-0.25 to 0.34	.759	Very similar post-March increases both years
Covid timeseries	7.22	7.09							
Control timeseries	7.39	6.63							
**MD: Capital**			2.27	-4.11 to 8.65	.482	0.31	-0.02 to 0.63	.066	Post-March decrease in 2019; Persistent rate in 2020
Covid timeseries	19.10	21.77							
Control timeseries	19.14	21.28							
**MD: Central**			-12.94	-39.11 to 13.23	.329	1.47	0.15 to 2.79	.029	Similar pre-March decrease both years; Steeper post-March increase in 2020
Covid timeseries	90.00	111.27							
Control timeseries	131.14	121.23							
**MD: Southern**			-0.21	-3.30 to 2.88	.895	0.23	0.05 to 0.40	.010	Similar pre-March decrease both years; New post-March increase in 2020
Covid timeseries	3.55	5.05							
Control timeseries	6.16	4.88							
**MD: Eastern Shore**			1.01	-5.08 to 7.10	.744	0.26	-0.13 to 0.66	.189	Graphical suggestion of steeper post-March increase in 2020
Covid timeseries	8.75	13.23							
Control timeseries	11.18	9.85							
**Washington, DC**			-8.20	-19.50 to 3.10	.153	-0.07	-0.62 to 0.48	.814	No steeper post-March increase in 2020
Covid timeseries^e^	45.94	49.07							
Control timeseries^f^	37.80	47.21							

^a^ CovidYear X AfterMarch interaction term in multivariable regression models.

^b^ CovidYear X AfterMarch X WeekSlope interaction term in multivariable regression models.

^c^ MD Covid timeseries: July 1, 2019 to September 7, 2020.

^d^ MD Control timeseries: July 2, 2018 to September 2, 2019.

^e^ DC Covid Timeseries: August 5, 2019 to October 25, 2020.

^f^ DC Control Timeseries: August 6, 2018 to October 20, 2019. MD = Maryland. PHOs = Public Health Orders. Washington DC models include quarter-year dummy variables to adjust for seasonality.

#### Maryland state and Maryland regions

Between July 2019 and March 2020, Maryland as a whole had a mean weekly nonfatal overdose count of 129.05. During this time, nonfatal overdoses were decreasing (Fig 2). After March 2020, mean weekly counts of nonfatal overdoses increased to 159.18. In contrast, mean weekly counts of nonfatal overdoses after March 2019 during the control time series decreased to 164.15 from a high of 175.41 before March. The five Maryland regions displayed differing patterns of change before and after March: the Capital region of Maryland had increases in nonfatal overdoses after March for both the Covid and control time series; the Western region had decreases after March for both time series; the Central, Southern, and Eastern Shore regions had increases after March during the Covid time series and decreases after March during the control time series.

After accounting for underlying trends, ITS models estimated an increase (not reaching statistical significance) of 22.5 nonfatal overdoses immediately following the PHOs in March during the Covid time series (B = 22.50; 95% CI, -2.97 to 48.00; p = .083). However, when comparing the difference between this change and the change after March during the control time series, we found no reliable differences between these mean-level changes (as reflected by the CovidYear X AfterMarch interaction term, B = -9.21; 95% CI, -47.56 to 29.15; p = .64). There were also no reliable differences between the mean-level changes before and after March for the Covid year versus the control time series for any of the five regions.

However, the pattern of change in the *slopes* of nonfatal overdoses after March—i.e., in the rate of change across weeks—was different during the Covid year than during the preceding control time series for Maryland as a whole; we estimate that there were an additional 2.4 nonfatal overdoses each week post March in 2020 compared with the post-March slope during the control period (B = 2.36; 95% CI, 0.65 to 4.06; p = .007). This statewide increase was driven by increases in specific subregions: Southern MD had an additional 0.2 overdoses each week post March in 2020 (B = 0.23; 95% CI, 0.05 to 0.40; p = .010) and Central MD had an additional 1.5 overdoses each week (B = 1.47; 95% CI, 0.15 to 2.79; p = .029). There was also some evidence for increases in the Capital region of MD compared to the control time series (B = 0.31; 95% CI, -0.02 to 0.63; p = .066). As shown in Fig 4A–4E, nonfatal overdoses in these regions were decreasing from July to March in both the Covid and control time series, but then diverged after March in region-specific ways: increasing in 2020 only (Southern MD), increasing more steeply in 2020 than in 2019 (Central MD), or not showing a subsequent decrease in 2020 after having decreased in 2019 (MD Capital region).

In the Eastern Shore region of MD, there was a graphical suggestion of a steeper post-March increase during the Covid-19 year, but week-to-week variability precluded its reaching statistical significance (B = 0.26; 95% CI, -0.13 to 0.66; p = .189). In the Western Shore region of MD, rates during the two years largely mirrored each other (B = 0.05; 95% CI, -0.25 to 0.34; p = .759).

#### Washington DC

In Washington DC, mean weekly counts of nonfatal overdoses increased after March for both the Covid and control timeseries. However, when comparing the difference between these changes for the two timeseries, we found no difference in mean-level changes immediately after March for the Covid versus control time series (B = -8.20; 95% CI, -19.50 to 3.10; p = .153), nor was there sign of a steeper post-March increase in slope during the Covid year (B = -0.07; 95% CI, -0.62 to 0.48; p = .814).

## Discussion

We found, after accounting for cyclical patterns and ongoing trends, that the Covid-19 pandemic and its accompanying PHOs were associated with steeper weekly growth in counts of suspected nonfatal opioid overdoses in many but not all regions for which we had data. To give one example in concrete terms, model-estimated weekly counts in one region (Central MD) increased from 116 to 127 in the period of March-September of 2019, and then increased from 81 to 142 in March-September of 2020. In contrast, for Washington, DC, the increases across those time periods were similar in 2019 (44 to 51) and 2020 (50 to 52).

One conclusion we can tentatively draw from our results is that they do not straightforwardly support the narrative that overdoses increased disproportionately in rural versus urban regions [33]. The geographical patterns in our data did not follow a tidy urban/rural dichotomy; however, there was some evidence that there were sustained increases in nonfatal overdoses in regions in MD with higher populations. Recent studies examining regional variation in overdoses before Covid-19 seem to suggest that large metropolitan counties have higher rates of fatal opioid overdoses compared to rural or micropolitan counties (Haffajee et al., 2019; Monnat, 2019). Even so, narrative reviews on geographical heterogeneity in opioid overdoses have found that rural/urban differences in opioid overdose rates appear to vary both between and within single and multi-state regions (e.g., western, northeastern, midwestern, or southern states) in the US (Rigg, Monnat, & Chavez, 2018 [34]), which may explain why changes were observed in Maryland, but not in its neighbor, Washington DC.

Before conducting our analyses, we suspected that differences in overdose rates across regions might be modulated simultaneously upward and downward by countervailing influences. Indeed, recent studies have documented a staggering increase in a variety of risk factors for substance use and overdose, including worsening psychiatric outcomes (e.g., stress, anxiety, depression), loneliness, economic distress, and disruptions to daily living and routine (e.g., childcare burden, poor sleep quality) [35–39], increased likelihood of using drugs alone, changes to illicit drug supplies [23], and reduced physical access to treatment services. On the other hand, mitigating influences could have included the relaxation of restrictions on dispensation of methadone and buprenorphine, disruption to supply chains for illicit drugs, and adherence to stay-at-home mandates and physical-distancing guidelines, which might reduce drug-acquisition behaviors [40–45].

Although we cannot be certain which factors were involved–or the relative contribution of each factor–in these changes in nonfatal overdoses, our findings suggest that within-state variability in changes in nonfatal overdoses during Covid-19 did occur and are likely to be found in other states as well [23]. Previous research has also found that the factors likely responsible for regional variation in opioid overdoses can vary geographically as well. For example, before Covid-19, economic distress appeared to have a stronger association with overdose mortality rates than opioid supply factors in rural counties in the US. In contrast, in urban counties, changes to opioid supply factors were more strongly associated with overdose mortality rates than economic distress factors (see Monnat, 2019). It is now known that the increases in *fatal* overdoses across the US during Covid-19 were primarily driven by synthetic opioids including illicitly manufactured fentanyl (CDC, 2020). It is possible that synthetic opioid supply/distribution changes in different regions in MD and Washington DC contributed to the differences in overdose numbers we observed.

Whatever the specific causes may be, the one clear conclusion we can draw from these findings is that regional variation is a paramount feature of both opioid overdose rates and factors contributing to opioid overdoses. Policymakers and public-health officials responsible for intervention design/deployment or legislation aimed at curtailing factors associated with overdoses in MD, Washington DC, and the US should keep this at the fore; it is likely that both policy and prevention will need to be tailored to specific regions. However, to more fully understand what factors contribute to regional differences, continual surveillance on a wide range of potential factors is needed. Continual follow-up analyses examining the mechanisms driving between and within region variation will need to identify high-risk areas, changes in factors driving overdose rates in these areas, and mechanisms to target via interventions or policy.

Finally, although our main goal was to isolate the impact of Covid-19 PHOs on nonfatal overdoses in Washington DC and Maryland, our use of a historical control timeseries of overdoses from previous years in the same geographical regions revealed potentially interesting findings about longer-term trends predating the Covid-19 pandemic and the impact that the pandemic may have had on these trends. Specifically, the steady long-term decline of nonfatal overdose in Maryland from when our control time series began, appeared to be decisively broken shortly after the implementation of the PHOs in March 2020. Further support for a trend change can only be inferred with more overdose data pre-July 2018 (the beginning of our control timeseries) and post-September 2020. Access to these data was limited by agreements with the original data sources. Future studies should examine longer-term trends in Maryland and elsewhere, because these findings may signal a protracted shift in risk for overdose which may need to be addressed by mitigation efforts.

### Limitations

There is an ambiguity inherent in our nonfatal-overdose data: overdoses that resulted in death were, in a sense, censored from the data set. Thus, we could not directly distinguish between an overdose that was avoided and an overdose that became fatal: either of those would be, in effect, one less nonfatal overdose. Ours is not the only published paper with that limitation [46]. However, the ambiguity is lessened by recent findings from the CDC and others: fatal opioid overdoses in Maryland and in Washington, DC, have indeed increased since the implementation of PHOs, over and above ongoing trends [23]. Thus, we assume that any flat or decreasing rates of nonfatal overdoses in our data are at least partly reflective of fatalities, though we cannot currently be precise about the proportions. We conceptualize our nonfatal-overdose data as a tally of an inherently undesirable event that increases the risk of future overdose events and places significant burden on healthcare systems [17]. Our study also only focuses on *counts* of nonfatal overdoses. While the data available to us precluded examination of *rates* of nonfatal overdose, future research should examine changes in rates of nonfatal overdoses before and during Covid-19 in MD and Washington DC.

## Conclusion

Our findings show that, for counts of suspected nonfatal overdoses, the *net* effect of the changes that accompanied the Covid-19 pandemic was deleterious in most geographical regions we assessed. However, some regions were clearly more affected than others. Monitoring nonfatal overdoses by region and factors known to contribute to regional variation in overdoses could be a valuable tool for identifying and understanding dangerous trends as the pandemic continues to evolve.

## Supporting information

S1 Appendix Methods, interrupted time series model results, and sensitivity analyses. (DOCX)
