# Supplementary material for: Nonfatal opioid overdoses before and after Covid-19: Regional variation in rates of change
Source: PLoS One. 2022 Mar 9;17(3):e0263893. doi: 10.1371/journal.pone.0263893 (PMC8906602; doi:10.1371/journal.pone.0263893)
Supplement: S1 Appendix — (DOCX) [file pone.0263893.s001.docx]

**S1 Appendix**

**eMethods**

**eTable 1.** Maryland Counties, Regional Membership, and Public Health Orders

**eTable 2.** Average Median Household Income and Average Population Estimates for the Five Maryland State Regions

**eTable 3.** Maryland State Interrupted Time Series Models for Nonfatal Overdoses after the Implementation of Covid-19 Public Health Orders

**eTable 4.** Washington DC Interrupted Time Series Models for Nonfatal Overdoses after the Implementation of Covid-19 Public Health Orders

**eTable 5.** Western Region (Maryland State) Interrupted Time Series Models for Nonfatal Overdoses after the Implementation of Covid-19 Public Health Orders

**eTable 6.** Capital Region (Maryland State) Interrupted Time Series Models for Nonfatal Overdoses after the Implementation of Covid-19 Public Health Orders

**eTable 7.** Central Region (Maryland State) Interrupted Time Series Models for Nonfatal Overdoses after the Implementation of Covid-19 Public Health Orders

**eTable 8.** Southern Region (Maryland State) Interrupted Time Series Models for Nonfatal Overdoses after the Implementation of Covid-19 Public Health Orders

**eTable 9.** Eastern Shore Region (Maryland State) Interrupted Time Series Models for Nonfatal Overdoses after the Implementation of Covid-19 Public Health Orders

**eTable 10.** Sensitivity Analyses: Maryland State Interrupted Time Series Models for Nonfatal Overdoses after the Implementation of Covid-19 Public Health Orders (20% reduction in weekly counts of nonfatal overdoses prior to July 2019)

**eTable 11.** Sensitivity Analyses: Maryland State Interrupted Time Series Models for Nonfatal Overdoses after the Implementation of Covid-19 Public Health Orders (25% reduction in weekly counts of nonfatal overdoses prior to July 2019)

This supporting material has been provided by the authors to give readers additional information about their work.

**eMethods**

**ODMAP**

The Overdose Detection Mapping Application Program (ODMAP) is an overdose data collection, visualization, and reporting platform developed by the Washington/Baltimore High Intensity Drug Trafficking Area program (HIDTA). The purpose of ODMAP is “to provide near real-time surveillance of suspected overdose events across jurisdictions in the United States of America (USA) and its territories, to support public safety and public health efforts to mobilize an immediate response to a sudden increase, or spike in overdose events” (ODMAP, 2020, p. 2). To accomplish this, ODMAP collects and stores geotagged data on suspected overdose events, which participating agencies upload through an API or enter manually. Authorized users can then visualize suspected overdoses that have been entered into the database via an online dashboard which can be used to filter the data by submission characteristics (e.g., suspected nonfatal vs. fatal overdose, naloxone administration) or run simple analyses. Some authorized users can also set-up automated overdose spike alerts within their jurisdiction or surrounding jurisdictions to enable public safety responses.

Data submission into ODMAP occurs by two methods: manual entry or through an Application Programming Interface (API). With manual entry, an authorized user utilizes their training and experience, evidence on scene, native agency protocols, and how the person is presenting themselves clinically to determine if the event is a suspected overdose (see case definition below). Once a suspected overdose is identified, ODMAP users utilize the ODMAP interface to enter the information into the system manually. Agencies who select an API solution, generally use the information from their records management software (RMS) due to it already serving as a centralized data repository for items such as incident reports, calls for service, or electronic patient care reports (eCPR). This process is accomplished programmatically using existing data and alleviates the burden of manual entry. The data fields within the ODMAP system are as follows:

Required Fields:

1. Date/Time
2. Location
3. Fatality status
4. Quantity of Naloxone administered

To enable agencies to collect a more robust dataset, ODMAP also allows users to submit the following optional data:

1. Case Number
2. Age
3. Gender
4. Primary Suspected Drug
5. Additional Suspected Drug(s)
6. Whether or not the victim was taken to the hospital
7. Whether or not the incident involved multiple victims
8. If a motor vehicle was involved
9. Who administered Naloxone

To control for duplicate submissions for a single suspected overdose within the system, ODMAP has a process in place to detect, alert, and delete such records. Once a submission is confirmed by the user, the system checks for any existing submissions that are within a 285 feet radius and one hour before or after one another. If a record is identified as a possible duplicate, one of the following will take place:

- For a manual entry versus any existing entry, the submitting user is alerted and given the opportunity to further investigate the existing data point prior to submitting a second entry.
- If an API submission is found to be a duplicate of an existing record, the API record is saved to ODMAP and then deleted, permitting it to be saved in the delete archive table for audit purposes.

It should also be noted if a submission is marked as a multiple victim overdose incident, it will override the system process.

More information about ODMAP, including operating policies and procedures, can be found at <http://www.odmap.org/>.

**Maryland and DC Health Data**

**Maryland Data.** The Maryland Institute for Emergency Medical Services Systems (MIEMSS) started data submission to ODMAP on July 1, 2018.

***Suspected nonfatal overdose definition.*** MIEMSS initial inclusion criteria for reporting a suspected opioid overdose to ODMAP consisted of any case in which a patient was administered naloxone by EMS or prior to EMS arrival. Per Maryland state legislative mandate, for any suspected opioid overdose, MIEMSS is required by law to submit the following data to ODMAP within 24-hours:

1. The date and time of the overdose
2. The approximate address where the overdose victim was initially encountered or where the overdose occurred
3. Whether an opioid overdose reversal drug was administered
4. Whether the overdose was fatal or nonfatal

*Data definition change of suspected overdoses.* To increase the specificity of suspected opioid overdoses, MIEMSS revised their inclusion criteria on July 1^st^, 2019. As a result of this data definition change, after June 30, 2019, MIEMSS only submitted data for an EMS case to ODMAP when:

1. Naloxone was administered and either:
   1. Primary impression of “Suspected opioid overdose (ICD-10-CM F11.9)” or “Poisoning/Overdose/Drug Abuse (ICD-10-CM T50.90)”, or
   2. Service-defined question (Do you think this patient is suffering from an opioid overdose?) response = Yes.

An analysis of the overdose data by MIEMSS revealed that this change resulted in a reduction of ~ 14.5% of suspected opioid overdose cases from the “naloxone only” methodology before July 1^st^, 2019. The additional reports from the naloxone only methodology likely represent cases in which naloxone was administered, but it was initially uncertain whether opioids contributed to the patient’s presentation.

**DC Health Data.** DC Health began sharing Emergency Medical Service (EMS) data with ODMAP on January 1, 2020. For the purpose of the analyses in this manuscript, DC Health also backfilled nonfatal overdose data from EMS from August 1, 2018 to December 31, 2019.

Data flows from a DC Health EMS repository to ODMAP in real time (on average within 15 minutes of a patient care record being closed).

***Suspected nonfatal overdose definition.*** A suspected nonfatal overdose is defined according to the National Emergency Medical Services Information System (NEMSIS) guidance. Standardized case definition is divided into primary and secondary elements.

*Primary elements (minimum elements required to identify a nonfatal overdose):*

A nonfatal opioid overdose is defined as any eligible 911 response where:

1. The Provider’s Primary Impression OR Provider’s Secondary Impression are opioid overdose related

OR

1. The Primary Symptom OR Other Associated Symptoms are opioid overdose related

OR

1. Medication Administered is naloxone or Narcan AND Response to Medication Administered is improved.

*Secondary elements:*

The Patient Care Report Narrative may be queried for opioid- AND overdose-related keywords in order to

validate previously identified incidents or identify incidents not previously included.

Cases are excluded if Type of Service Requested is a non-emergency response or Initial Patient Acuity is “Dead Without Resuscitation Efforts”.

Data selections for Provider’s Primary Impression and Provider’s Secondary Impression and selections for Primary Symptom and Other Associated Symptoms are mapped on the backend to codes from the International Classification of Diseases, Tenth Revision Clinical Modification (ICD-10-CM). These include “T-codes” which indicate injury, poisoning, and certain other consequences of external causes (e.g., T40.0, T40.1, T40.6), and/or “F-codes” (e.g., F11.9, F11). See https://nasemso.org/wp-content/uploads/EMS-Nonfatal-Opioid-Overdose-Case-Definition_V5.pdf for a complete list of the ICD-10-CM codes.

**Nonfatal Overdoses: Sensitivity and Specificity**

It is difficult to quantify how many opioid overdoses may not result in calls to EMS, public health, or law enforcement. However, previous research quantifying the frequency in which EMS medical personal were called in response to an overdose suggest that EMS medical personal respond to an overdose approximately 46 – 66% of the time (Darke, Mattick, & Degenhardt, 2003; Pollini, McCall, Mehta, Vlahov, & Strathdee, 2006; Sherman, Cheng, & Kral, 2007). Furthermore, for a sample of participants in and around Baltimore, Maryland, 33.3% of individuals who overdosed went to an ER for treatment.

**Interrupted Time Series Analysis**

**Data Pre-Processing.** We first aggregated the Maryland and DC daily nonfatal datasets to the weekly level.

To rule-out normative year-to-year changes in overdoses and history effects, we split the MD dataset into two timeseries: (a) a COVID-19 timeseries (July 1, 2019 to September 7, 2020), which we designated the ”Covid year” and which contained weeks before and after the implementation of public health orders in MD state, and (b) a no-treatment historical control timeseries (July 2, 2018 to September 2, 2019) which contained data on overdoses immediately preceding the COVID-19 timeseries. We differentiated each timeseries with an indicator variable (1 = COVID-19 timeseries; 0 = historical control timeseries).

For the DC dataset, we split the data into a COVID-19 timeseries (August 5, 2019 to October 25, 2020) and a no-treatment historical control timeseries (August 6, 2018 to October 20, 2019). We differentiated each timeseries with an indicator variable (1 = COVID-19 timeseries).

To correct for the EMS data definition change that occurred on July 1^st^ 2019 in Maryland state, we subtracted 14.5% off of each week’s final reported number of nonfatal overdoses from July 2^nd^ 2018 – June 30^th^ 2019.

**ITS models.** We estimated the following regression equation for the MD state, MD regional, and Washington DC datasets:

${NonFatalODs}_{w}= \beta_{0}+ \beta_{1}{Week}_{w}+ \beta_{2}{PHO}_{t}+ \beta_{3}{CovidTS}_{t}+ \beta_{4}{Week\_PHO}_{t}+ \beta_{5}{Week\_CovidTS}_{t}+ \beta_{6}{CovidTS\_PHO}_{t}+ \beta_{7}{Week\_CovidTS\_PHO}_{t}+ \varepsilon_{w}$

Where *NonFatalODs* is the nonfatal overdose count at week *w, Week* is the number of weeks since the beginning of the analysis period, *PHO* is a dummy variable specifying pre- or post-Covid public health order implementation (March 31^st^), *CovidTS* is a dummy variable specifying the Covid timeseries (t = 1) or historical control timeseries (t = 0), *Week_PHO* is the number of weeks since the implementation of the Covid-19 public health orders, *Week_CovidTS* is the number of weeks since the beginning of the Covid timeseries, *CovidTS_PHO* is a dummy variable specifying pre- or post-Covid public health order implementation for the Covid-19 timeseries, and *Week_CovidTS_PHO* is the number of weeks since the implementation of the Covid-19 public health orders for the Covid-19 timeseries.

Although Maryland and DC declared a state of emergency at different times in early March, both regions implemented stay-at-home orders on the same day (March 30, 2020). Thus, we made the assumption that changes related to the PHOs (e.g., disruption to addiction treatment services, changes to illicit drug supplies, loneliness/boredom, childcare/economic burden) would have delayed effects on overdose rates, and selected March 31, 2020 as the intervention “change point.”

We also tested the inclusion of quarter-yearly indicator variables to adjust for seasonal changes in nonfatal overdoses each year. These indicator variables only improved the relative fit of the Washington DC models as measured by the AIC and BIC. Thus, we included these indicators in the Washington DC models and excluded them from all Maryland models. All models used heteroskedasticity-and-autocorrelation-consistent standard errors with the lag selected according to Newey and West (1994) using the NeweyWest function in the sandwich package (version 2.5-1)(Zeileis, 2004) in R (R Core Team, 2020). All models were fit using R version 3.6.2.

**MD Data Definition Change: Sensitivity Analyses**

To examine the robustness of the ITS models to misspecifications in the estimates of the impact of the MIEMSS revised suspected overdose inclusion criteria on July 1^st^, 2019, we fit additional ITS models for MD state subtracting 5, 10, 20, or 25% from weekly counts of nonfatal overdoses in Maryland prior to July 2019. For all models excluding the models with a 25% reduction in weekly counts of nonfatal overdoses prior to July 2019, we found significantly steeper post-March increases in nonfatal overdoses during the Covid-19 year compared to the control time series. The parameter estimates from the models with 20 and 25% reduction in weekly counts of nonfatal overdoses prior to July 2019 are displayed in eTables 9 and 10.

**eTable 1. Maryland Counties, Regional Membership, and Public Health Orders**

| **County** | **Region** | **Stay-at-Home Orders** | **Ban on Gatherings > 1 – 10 People** |
| --- | --- | --- | --- |
| Garrett County | Western | 3/30/2020 | 3/19/2020 |
| Allegany County | Western | 3/30/2020 | 3/19/2020 |
| Washington County | Western | 3/30/2020 | 3/19/2020 |
| Frederick County | Capital | 3/30/2020 | 3/19/2020 |
| Montgomery County | Capital | 3/30/2020 | 3/19/2020 |
| Prince George's County | Capital | 3/30/2020 | 3/19/2020 |
| Anne Arundel County | Central | 3/30/2020 | 3/19/2020 |
| Baltimore City | Central | 3/30/2020 | 3/19/2020 |
| Baltimore County | Central | 3/30/2020 | 3/19/2020 |
| Carroll County | Central | 3/30/2020 | 3/19/2020 |
| Harford County | Central | 3/30/2020 | 3/19/2020 |
| Howard County | Central | 3/30/2020 | 3/19/2020 |
| Calvert County | Southern | 3/30/2020 | 3/19/2020 |
| Charles County | Southern | 3/30/2020 | 3/19/2020 |
| St. Mary's County | Southern | 3/30/2020 | 3/19/2020 |
| Kent County | Eastern Shore | 3/30/2020 | 3/19/2020 |
| Queen Anne's County | Eastern Shore | 3/30/2020 | 3/19/2020 |
| Talbot County | Eastern Shore | 3/30/2020 | 3/19/2020 |
| Caroline County | Eastern Shore | 3/30/2020 | 3/19/2020 |
| Dorchester County | Eastern Shore | 3/30/2020 | 3/19/2020 |
| Wicomico County | Eastern Shore | 3/30/2020 | 3/19/2020 |
| Somerset County | Eastern Shore | 3/30/2020 | 3/19/2020 |
| Worcester County | Eastern Shore | 3/30/2020 | 3/19/2020 |
| Cecil County | Eastern Shore | 3/30/2020 | 3/19/2020 |

*Note.* Timing of stay-at-home orders and gathering bans were obtained from <https://data.cdc.gov/Policy-Surveillance/U-S-State-Territorial-and-County-Stay-At-Home-Orde/qz3x-mf9n/data> and <https://data.cdc.gov/Policy-Surveillance/U-S-State-and-Territorial-Gathering-Bans-March-11-/7xvh-y5vh/data>

**eTable 2. Average Median Household Income and Average Population Estimates for the Five Maryland State Regions**

|  | **Capital Region** | **Central Region** | **Eastern Shore Region** | **Southern Region** | **Western Region** | **p-value** |
| --- | --- | --- | --- | --- | --- | --- |
| Median Household Income (Mean) | 97,156.67 | 89,186.50 | 63,986.56 | 99,720.33 | 53,123.33 | 0.018 |
| Population (Mean) | 739,854.00 | 458,278.67 | 50,741.67 | 123,097.33 | 83,493.00 | 0.001 |

*Note.* Median household income data for MD counties were obtained from the American Community Survey 5-year data (2019) from census.gov. MD county population estimates for 2019 were obtained from the Census Bureau’s Population Estimates Program (PEP) from Census.gov. P-values are derived from omnibus Kruskal-Wallis tests.

**eTable 3. Maryland State Interrupted Time Series Models for Nonfatal Overdoses after the Implementation of Covid-19 Public Health Orders**

|  | **Estimate** | **95% CI** | **p-value** |
| --- | --- | --- | --- |
| Intercept | 228.42 | 208.87, 247.97 | < .001 |
| Week Slope | - 2.65 | -3.36, -1.95 | < .001 |
| Public Health Orders (PHOs) | 31.73 | 13.99, 49.47 | < .001 |
| Covid Time Series (TS) | - 61.45 | -93.92, -26.22 | < .001 |
| Week Slope x PHOs | 3.29 | 1.85, 4.73 | < .001 |
| Week Slope x Covid TS | 0.80 | -0.47, 2.08 | 0.216 |
| Covid TS x PHOs | - 9.21 | -47.56, 29.15 | 0.635 |
| Week Slope x Covid TS x PHOs | 2.36 | 0.65, 4.06 | 0.007 |

**eTable 4. Washington DC Interrupted Time Series Models for Nonfatal Overdoses after the Implementation of Covid-19 Public Health Orders**

|  | **Estimate** | **95% CI** | **p-value** |
| --- | --- | --- | --- |
| Intercept | 45.23 | 34.05, 56.42 | < .001 |
| Week Slope | -0.33 | -0.72, 0.05 | 0.089 |
| Public Health Orders (PHOs) | 16.32 | -5.76, 38.41 | 0.146 |
| Covid Time Series (TS) | 6.06 | -2.41, 14.54 | 0.159 |
| Week Slope x PHOs | 0.26 | -0.50, 1.02 | 0.496 |
| Week Slope x Covid TS | 0.11 | -0.23, 0.45 | 0.518 |
| Covid TS x PHOs | -8.20 | -19.50, 3.10 | 0.153 |
| Week Slope x Covid TS x PHOs | -0.07 | -0.62, 0.48 | 0.814 |

**Notes.** Models adjusted for quarter-yearly seasonality.

**eTable 5. Western Region (Maryland State) Interrupted Time Series Models for Nonfatal Overdoses after the Implementation of Covid-19 Public Health Orders**

|  | **Estimate** | **95% CI** | **p-value** |
| --- | --- | --- | --- |
| Intercept | 8.01 | 6.87, 9.15 | < .001 |
| Week Slope | -0.03 | -0.07, 0.01 | 0.107 |
| Public Health Orders (PHOs) | -2.95 | -4.32, -1.58 | < .001 |
| Covid Time Series (TS) | 0.26 | -1.66, 2.19 | 0.788 |
| Week Slope x PHOs | 0.27 | 0.18, 0.37 | < .001 |
| Week Slope x Covid TS | -0.02 | -0.08, 0.04 | 0.522 |
| Covid TS x PHOs | 0.73 | -2.80, 4.27 | 0.682 |
| Week Slope x Covid TS x PHOs | 0.05 | -0.25, 0.34 | 0.759 |

**eTable 6. Capital Region (Maryland State) Interrupted Time Series Models for Nonfatal Overdoses after the Implementation of Covid-19 Public Health Orders**

|  | **Estimate** | **95% CI** | **p-value** |
| --- | --- | --- | --- |
| Intercept | 17.81 | 16.03, 19.59 | < .001 |
| Week Slope | 0.07 | -0.00, 0.14 | 0.067 |
| Public Health Orders (PHOs) | 2.05 | -2.63, 6.73 | 0.388 |
| Covid Time Series (TS) | 3.43 | -0.60, 7.46 | 0.094 |
| Week Slope x PHOs | -0.17 | -0.43, 0.09 | 0.204 |
| Week Slope x Covid TS | -0.17 | -0.31, -0.03 | 0.019 |
| Covid TS x PHOs | 2.27 | -4.11, 8.65 | 0.482 |
| Week Slope x Covid TS x PHOs | 0.31 | -0.02, 0.63 | 0.066 |

**eTable 7. Central Region (Maryland State) Interrupted Time Series Models for Nonfatal Overdoses after the Implementation of Covid-19 Public Health Orders**

|  | **Estimate** | **95% CI** | **p-value** |
| --- | --- | --- | --- |
| Intercept | 179.32 | 163.81, 194.83 | < .001 |
| Week Slope | -2.41 | -3.05, -1.77 | < .001 |
| Public Health Orders (PHOs) | 29.81 | 17.02, 42.60 | < .001 |
| Covid Time Series (TS) | -58.74 | -86.00, -31.49 | < .001 |
| Week Slope x PHOs | 2.93 | 1.91, 3.96 | < .001 |
| Week Slope x Covid TS | 0.92 | -0.17, 2.01 | 0.098 |
| Covid TS x PHOs | -12.94 | -39.11, 13.23 | 0.329 |
| Week Slope x Covid TS x PHOs | 1.47 | 0.15, 2.79 | 0.029 |

**eTable 8. Southern Region (Maryland State) Interrupted Time Series Models for Nonfatal Overdoses after the Implementation of Covid-19 Public Health Orders**

|  | **Estimate** | **95% CI** | **p-value** |
| --- | --- | --- | --- |
| Intercept | 8.25 | 7.26, 9.25 | < .001 |
| Week Slope | -0.10 | -0.16, -0.05 | < .001 |
| Public Health Orders (PHOs) | 1.01 | -1.10, 3.12 | 0.344 |
| Covid Time Series (TS) | -2.84 | -5.67, -0.02 | 0.049 |
| Week Slope x PHOs | 0.08 | -0.15, 0.30 | 0.490 |
| Week Slope x Covid TS | 0.01 | -0.10, 0.12 | 0.803 |
| Covid TS x PHOs | -0.21 | -3.30, 2.88 | 0.895 |
| Week Slope x Covid TS x PHOs | 0.23 | 0.05, 0.40 | 0.010 |

**eTable 9. Eastern Shore Region (Maryland State) Interrupted Time Series Models for Nonfatal Overdoses after the Implementation of Covid-19 Public Health Orders**

|  | **Estimate** | **95% CI** | **p-value** |
| --- | --- | --- | --- |
| Intercept | 14.63 | 12.54, 16.72 | < .001 |
| Week Slope | -0.17 | -0.27, -0.07 | < .001 |
| Public Health Orders (PHOs) | 1.72 | -1.84, 5.28 | 0.341 |
| Covid Time Series (TS) | -3.56 | -6.65, -0.47 | 0.024 |
| Week Slope x PHOs | 0.19 | -0.07, 0.45 | 0.144 |
| Week Slope x Covid TS | 0.06 | -0.07, 0.19 | 0.365 |
| Covid TS x PHOs | 1.01 | -5.08, 7.10 | 0.744 |
| Week Slope x Covid TS x PHOs | 0.26 | -0.13, 0.66 | 0.189 |

**eTable 10. Sensitivity Analyses: Maryland State Interrupted Time Series Models for Nonfatal Overdoses after the Implementation of Covid-19 Public Health Orders (20% reduction in weekly counts of nonfatal overdoses prior to July 2019)**

|  | **Estimate** | **95% CI** | **p-value** |
| --- | --- | --- | --- |
| Intercept | 213.73 | 195.44, 232.02 | < .001 |
| Week Slope | - 2.48 | -3.14, -1.82 | < .001 |
| Public Health Orders (PHOs) | 26.01 | 8.53, 43.49 | 0.004 |
| Covid Time Series (TS) | - 46.76 | -81.62, -11.90 | 0.009 |
| Week Slope x PHOs | 3.80 | 2.31, 5.30 | < .001 |
| Week Slope x Covid TS | 0.63 | -0.62, 1.88 | 0.320 |
| Covid TS x PHOs | - 3.48 | -41.98, 35.02 | 0.858 |
| Week Slope x Covid TS x PHOs | 1.85 | 0.16, 3.54 | 0.033 |

**eTable 11. Sensitivity Analyses: Maryland State Interrupted Time Series Models for Nonfatal Overdoses after the Implementation of Covid-19 Public Health Orders (25% reduction in weekly counts of nonfatal overdoses prior to July 2019)**

|  | **Estimate** | **95% CI** | **p-value** |
| --- | --- | --- | --- |
| Intercept | 200.37 | 182.78, 217.95 | < .001 |
| Week Slope | - 2.33 | -2.94, -1.71 | < .001 |
| Public Health Orders (PHOs) | 20.80 | 3.17, 38.43 | 0.021 |
| Covid Time Series (TS) | - 33.40 | -67.74, 0.94 | 0.056 |
| Week Slope x PHOs | 4.26 | 2.65, 5.88 | < .001 |
| Week Slope x Covid TS | 0.48 | -0.75, 1.70 | 0.442 |
| Covid TS x PHOs | 1.72 | -37.10, 40.51 | 0.930 |
| Week Slope x Covid TS x PHOs | 1.38 | -0.35, 3.12 | 0.116 |

**References**

Darke, S., Mattick, R. P., & Degenhardt, L. (2003). The ratio of non‐fatal to fatal heroin overdose. *Addiction*, *98*(8), 1169–1171.

ODMAP: Overdose Detection Mapping Application Program. (2020). *Overdose Detection Mapping Application Program Research Guidance and Procedures.* Retrieved from <http://www.odmap.org/Content/docs/ODMAP-Research-Guidelines.pdf>

Pollini, R. A., McCall, L., Mehta, S. H., Vlahov, D., & Strathdee, S. A. (2006). Non-fatal overdose and subsequent drug treatment among injection drug users. *Drug and Alcohol Dependence*, *83*(2), 104–110.

R Core Team (2020). R: A language and environment for statistical computing. R Foundation for Statistical Computing, Vienna, Austria. URL <https://www.R-project.org/>.

Sherman, S. G., Cheng, Y., & Kral, A. H. (2007). Prevalence and correlates of opiate overdose among young injection drug users in a large US city. *Drug and Alcohol Dependence*, *88*(2–3), 182–187.

Zeileis A (2004). “Econometric Computing with HC and HAC Covariance Matrix Estimators.” Journal of Statistical Software, 11(10), 1–17. doi: 10.18637/jss.v011.i10.
